# Monitoring the haemodynamic response to visual stimulation in glaucoma patients

**DOI:** 10.1038/s41598-021-92857-x

**Published:** 2021-06-30

**Authors:** R. Re, D. Messenio, G. Marano, L. Spinelli, I. Pirovano, D. Contini, R. Colombo, P. Boracchi, E. Biganzoli, R. Cubeddu, A. Torricelli

**Affiliations:** 1 Dipartimento di Fisica, Politecnico di Milano, Piazza Leonardo da Vinci 32, 20133 Milan, Italy; 2 Istituto di Fotonica e Nanotecnologie, Consiglio Nazionale delle Ricerche, Piazza Leonardo da Vinci 32, 20133 Milan, Italy; 3 Department of Clinical Sciences, Eye Clinic, ASST Fatebenefratelli Sacco Hospital, University of Milan, Milan, Italy; 4 Laboratorio di Statistica Medica, Biometria ed Epidemiologia “G.A. Maccacaro”, Dipartimento di Scienze Cliniche e di Comunità, Università degli Studi di Milano, Via Vanzetti 5, Milan, Italy; 5 Istituto di Tecnologie Biomediche, Consiglio Nazionale delle Ricerche, via Fratelli Cervi 93, 20090 Segrate, MI Italy; 6 Unità di Statistica Medica, Biometria e Bioinformatica, Fondazione IRCCS Istituto Nazionale dei Tumori di Milano, Via Vanzetti 5, Milan, Italy

**Keywords:** Brain imaging, Neurological disorders, Near-infrared spectroscopy

## Abstract

In this paper, we used time-domain functional near infrared spectroscopy (TD-fNIRS) to evaluate the haemodynamic response function (HRF) in the occipital cortex following visual stimulation in glaucomatous eyes as compared to healthy eyes. A total of 98 subjects were enrolled in the study and clinically classified as healthy subjects, glaucoma patients (primary open-angle glaucoma) and mixed subjects (i.e. with a different classification for the two eyes). After quality check data were used from HRF of 73 healthy and 62 glaucomatous eyes. The amplitudes of the oxygenated and deoxygenated haemoglobin concentrations, together with their latencies with respect to the stimulus onset, were estimated by fitting their time course with a canonical HRF. Statistical analysis showed that the amplitudes of both haemodynamic parameters show a significant association with the pathology and a significant discriminating ability, while no significant result was found for latencies. Overall, our findings together with the ease of use and noninvasiveness of TD-NIRS, make this technique a promising candidate as a supporting tool for a better evaluation of the glaucoma pathology.

## Introduction

The glaucoma is a multifactorial optic neuropathy characterized by a progressive loss of retinal ganglion cells, changes in optic disk morphology and visual field defects^1^. Intraocular pressure (IOP) is a recognized risk factor for the development and progression of glaucomatous damage^2^. The focus of glaucoma treatment has been aimed at lowering IOP, currently the only modifiable risk factor. Unfortunately, the reduction of IOP is not sufficient to slow or stop the progression of the disease, which causes irreversible visual loss^3^. This fact demonstrates that IOP alone does not explain the pathogenesis of this complex neurodegenerative disease, that has important analogies with other neurodegenerative pathologies such as Alzheimer’s disease^4^.

Experimental and human studies have shown that the glaucoma-associated neurodegeneration process is not limited to the optic nerve, but involves the whole visual pathway extending to the lateral geniculate nucleus (LGN)^5,6^ up to the primary visual cortex (V1)^7,8^. Indeed, the optic nerve is considered a portion of white matter of the central nervous system. In 1993 the first histological (autoptic) study detecting a significant reduction of LGN magnocellular cells in glaucomatous subjects was performed^9^. In 2006 Gupta et al*.*, in a case report, showed degenerative changes in human visual cortex (cortical ribbon thickness reduction) and reduced LGN cells size^10^.

The relationships between anatomical (morphological) and functional evaluations in glaucoma are of fundamental importance to quantify the damage of the disease. To this purpose, Gupta stressed that “a multidisciplinary approach is needed to better understand glaucomatous pathology in the central nervous system at various stages of disease”^11^.

Different researchers employed functional magnetic resonance imaging (fMRI), based on the blood oxygenation level-dependent (BOLD) signal evaluation^12^, to study the functional cerebral involvement in glaucomatous patients. In particular, Lestak et al*.* found alterations of fMRI during checkboard stimulation^13^: Subjects with advanced glaucoma showed visual field alteration in corresponding locations of the flattened cortex^14^.

Recently, the use of functional near infrared spectroscopy (fNIRS) was also proposed for studying glaucoma pathology^15^, by exploiting the possibility to noninvasively monitor functional changes in oxygenated haemoglobin (O_2_Hb) and deoxygenated haemoglobin (HHb) concentration in brain cortex. The haemodynamic response of the visual cortex in 8 glaucoma subjects showed a lower change in O_2_Hb concentration with respect to the control ones.

Despite these preliminary studies, the relationship between glaucoma and haemodynamic response function in the cerebral cortex has not been fully explored and understood.

As a preliminary step for the investigation of the morpho-functional involvement of the occipital brain structures (visual areas) in patients with chronic simple glaucoma, healthy patients and mixed ones (with only one glaucomatous eye), we evaluated whether the hemodynamic responses evoked by stimulating each eye are different between glaucomatous and healthy eyes. This could provide a first evidence to promote the assessment of the relationships between fNIRS parameters, morphological parameters and glaucoma disease. The aim of our work is to evaluate whether there is a reduction in the cortical haemodynamic response in glaucomatous eyes with respect to the healthy ones. To this purpose we have used fNIRS in the time domain (TD)^16^ modality. TD-fNIRS has the advantage over conventional fNIRS approaches of an improved depth selectivity, allowing an easier removal of movement artefacts and possible physiological contamination in the signal originating from the capillary bed in the superficial tissues (scalp and skull) of the head. Further, TD-fNIRS yields absolute estimates for O_2_Hb and HHb concentrations in the brain cortex.

## Results

### Post-acquisition subjects’ classification

After data acquisition from the 94 subjects, we excluded 8 subjects for different reasons: 1 subject was not collaborative; 2 had a wrong optodes positioning on the head, which was observed only after the acquisition; 3 did not pass the TD-fNIRS quality check; in 2 subjects, with head circumference > 61 cm, we could not place the EEG cap in the right position. The final number of subjects included in the statistical analysis was 86. Their demographic data are listed in Supplementary Table ST1. On the basis of the standard clinical exams, the subjects were classified as: 31 NORM, 43 GLAUCOMA, and 12 “mixed subjects”, where the two eyes did not have the same classification.

### Eye’s classification

Since the acquisitions were accomplished with one eye at a time and the glaucoma could be present with a different stage of progression in each eye, for each subject we considered the single eye and the single hemisphere. We had a total of 172 eyes and 344 hemispheres. After performing the signal’s quality check, 168 eyes and 336 hemispheres were left. The eyes were clinically classified as: 73 NORM and 95 GLAUCOMA.

### TD-fNIRS: baseline optical properties and time courses of haemodynamic parameters

The baselines optical properties, μ_a_ and μ_s_^’^ at 687 and 826 nm for the occipital brain cortex, are presented in the Table 1 averaged over the two hemispheres (O1 and O2 positions^17^). These values were calculated as an average during the initial 30 s of the acquisition, which correspond to the baseline period of the measurement protocol, i.e. no visual stimuli are presented to the subject. No significant difference was found between healthy subjects and glaucoma patients.

**Table 1:** Optical properties (average ± standard deviation over subjects and positions) in healthy subjects (NORM) and patients (GLAUCOMA) at 687 nm and 826 nm.

	μ_a_ (cm^−1^)		μ_s_′ (cm^−1^)	
	687 nm	826 nm	687 nm	826 nm
NORM	0.15 ± 0.04	0.14 ± 0.03	10.0 ± 1.5	8.7 ± 1.4
GLAUCOMA	0.13 ± 0.04	0.13 ± 0.03	9.9 ± 1.8	8.7 ± 1.6

*μ*_*a*_ absorption coefficient_;_ *μ*_*s*_′ scattering coefficient.

**Figure 1:**
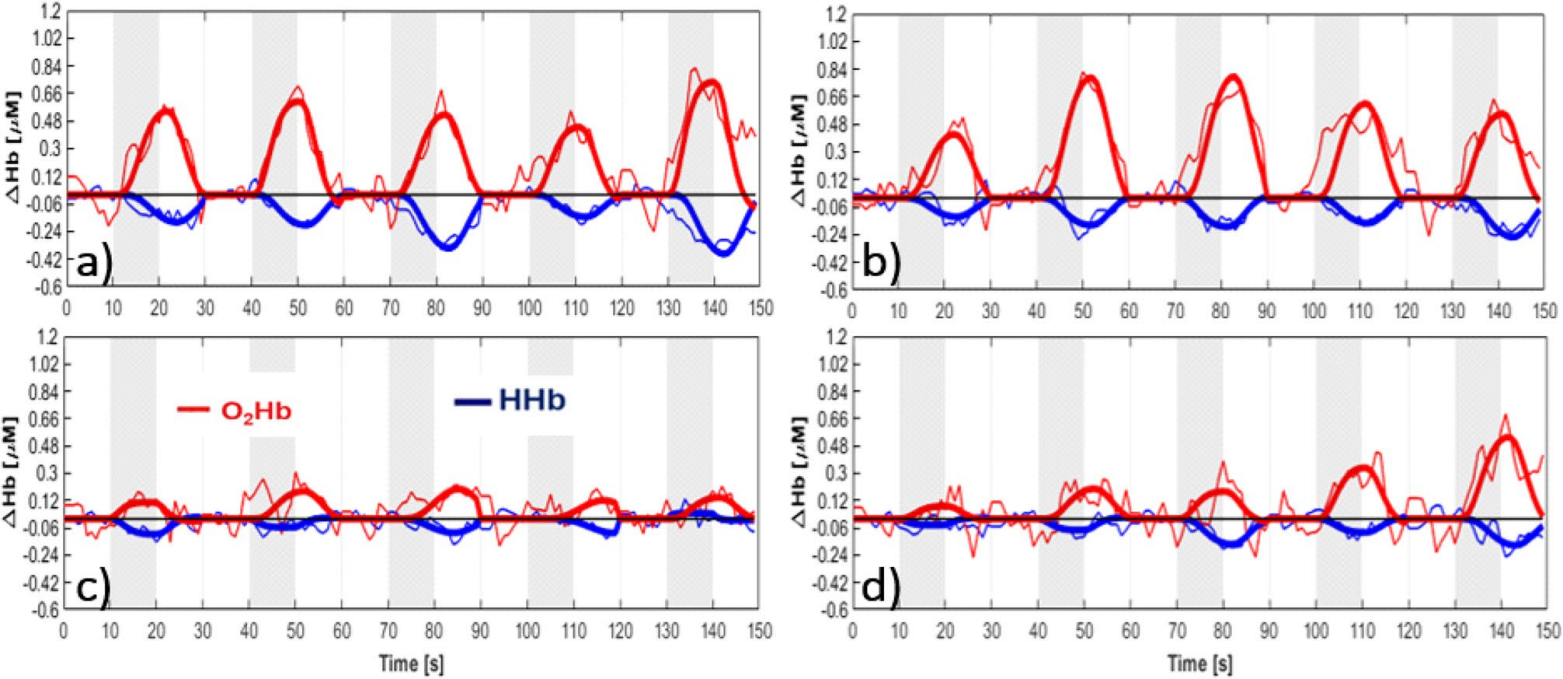
Typical TD-fNIRS time courses (thin line) for O_2_Hb (red) and HHb (blue) concentration changes and corresponding fitting with the HRF (thick lines) for a control subject (**a,b**) and a glaucoma patient (**c,d**) in the left (**a–c**) and right (**b–d**) hemisphere. The results are shown for the stimulation of the right eye. The stimuli periods are represented in grey.

The typical TD-fNIRS time courses, expressed as variations with respect to the baseline, obtained for O_2_Hb and HHb concentrations are shown in Fig. 1 for the two hemispheres when the subject is fixing the screen with the right eye. In the NORM subject (a,b), as expected, we can observe the canonical increase in O_2_Hb concentration accompanied by a decrease in HHb concentration each time a stimulus is presented. Conversely, for the GLAUCOMA patient (c,d) the fNIRS signal appears strongly reduced. The median and quartiles (Q1, Q3) for the amplitude A of the HRF for NORM and GLAUCOMA subjects were respectively: 0.65 (0.45, 1.13) μM and 0.41 (0.18, 0.60) μM for A_O2Hb_; − 0.24 (− 0.38, − 0.16) μM and − 0.14 (− 0.21, − 0.06) μM for A_HHb_. The median and quartiles (Q1, Q3) for the delay τ of the HRF for NORM and GLAUCOMA subjects were respectively: 5.0 (4.2, 5.9) s and 4.0 (3.1, 5.7) s for τ_O2Hb_; 5.2 (4.6, 5.6) and 4.6 (3.7, 5.6) s for τ_HHb_. Median and quartiles (Q1, Q3) data for eyes and hemispheres are reported in Supplementary Table ST2. We excluded from further analysis 41 stimulation cycles for which τ > 20 s, therefore longer than the stimulation period.

### Statistical analysis

**Figure 2:**
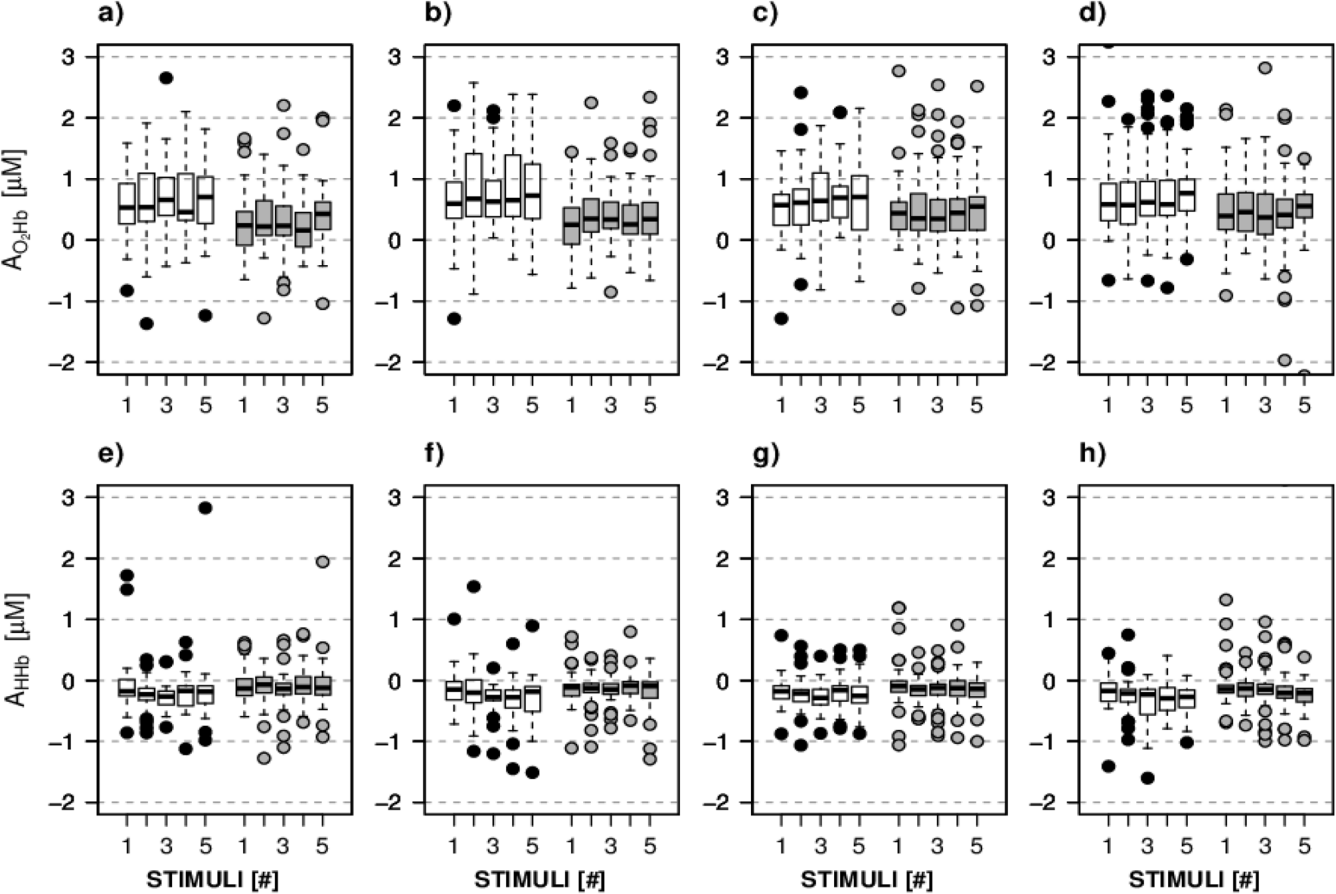
Distribution of A_O2Hb_ (**a–d**) and A_HHb_ (**e–h**) over the 5 stimuli for healthy eyes (NORM, white) and glaucomatous eyes (GLAUCOMA, grey). (**a,e**) Left eye, left hemisphere; (**b,f**) left eye, right hemisphere; (**c,g**) right eye, left hemisphere; (**d,h**) right eye, right hemisphere.

**Figure 3:**
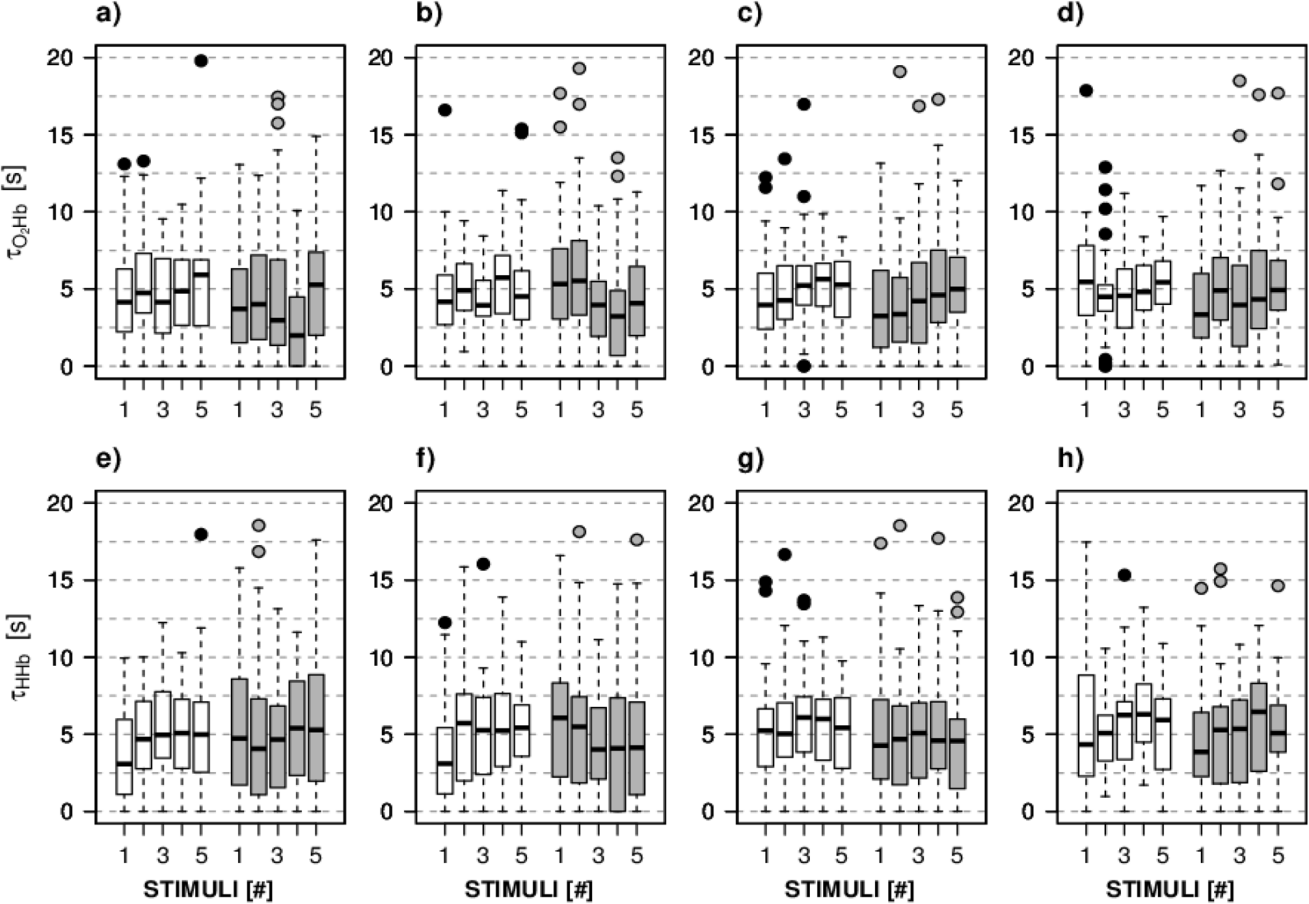
Distribution of τ_O2Hb_ (**a–d**) and τ_HHb_ (**e–h**) over the 5 stimuli for healthy eyes (NORM, white) and glaucomatous eyes (GLAUCOMA, grey). (**a,e**) Left eye, left hemisphere; (**b,f**) left eye, right hemisphere; (**c,g**) right eye, left hemisphere; (**d,h**) right eye, right hemisphere.

The distributions of A and τ parameters for O_2_Hb and HHb as obtained from the HRF fitting are shown in Figs. 2 and 3, respectively; corresponding medians and quartiles are reported in Supplementary Table ST2. The distributions do not show any clear pattern related to the combinations of experimental factors. Specifically, we do not report a trend across the five stimuli. Some differences between glaucomatous and healthy eyes are shown for the distributions of peak amplitudes: in particular, the medians of such distributions are closer to 0 μM for glaucomatous eyes as compared to healthy eyes. This pattern is more evident for A_O2Hb_ with respect to A_HHb_. In the following subsections, the results from the discriminant ability and association’s analysis are reported.

#### HRF amplitudes and latencies recorded at single hemisphere

For each parameter recorded at single hemisphere, we evaluated its relationship with the pathological classification. This was obtained by estimating 16 distinct logistic models including a single independent variable (Table 2). For each parameter, four tests were performed, thus all the C.I.s and the p-values were corrected for such multiplicity.

**Table 2:** Association and discriminant ability of TD-fNIRS parameters recorded at single hemisphere.

TD-fNIRS parameters	OR Est (95% C.I.)	Wald test			c index Est (95% C.I.)
		Df	χ^2^	p-value	
**τ**_**HHb**_					
Left hemisphere	1.05 (0.90, 1.23)	1	0.614	> 0.999	0.513 (0.400, 0.626)
Right hemisphere	0.93 (0.81, 1.07)	1	1.600	0.8220	0.555 (0.442, 0.668)
Ipsilateral hemisphere	1.01 (0.89, 1.15)	1	0.036	> 0.999	0.524 (0.410, 0.623)
Contralateral hemisphere	0.97 (0.85, 1.10)	1	0.450	> 0.999	0.548 (0.435, 0.660)
**τ**_**O2Hb**_					
Left hemisphere	0.89 (0.73, 1.08)	1	2.180	0.5592	0.586 (0.477, 0.696)
Right hemisphere	0.98 (0.81, 1.20)	1	0.046	> 0.999	0.532 (0.421, 0.643)
Ipsilateral hemisphere	0.92 (0.75, 1.12)	1	1.110	> 0.999	0.595 (0.486, 0.704)
Contralateral hemisphere	0.97 (0.84, 1.13)	1	0.228	> 0.999	0.529 (0.418, 0.640)
**A**_**HHb**_					
Left hemisphere	3.31 (0.35, 31.51)	1	1.770	0.7322	0.677 (0.503, 0.851)
Right hemisphere	12.63 (1.35, 117.74)	1	8.130	0.0174*	0.707 (0.517, 0.897)
Ipsilateral hemisphere	6.94 (0.95, 50.59)	1	6.000	0.0574	0.687 (0.508, 0.866)
Contralateral hemisphere	3.71 (0.78, 15.58)	1	4.470	0.1379	0.694 (0.511, 0.877)
**A**_**O2Hb**_					
Left hemisphere	0.32 (0.11, 0.95)	1	6.890	0.0348*	0.691 (0.587, 0.795)
Right hemisphere	0.45 (0.23, 0.89)	1	8.490	0.0143*	0.699 (0.597, 0.802)
Ipsilateral hemisphere	0.53 (0.31, 0.89)	1	9.310	0.0091**	0.692 (0.589, 0.795)
Contralateral hemisphere	0.45 (0.21, 0.99)	1	6.390	0.0458*	0.695 (0.592, 0.798)

Results obtained by estimating 16 distinct logistic models including a single independent variable. Each p-value and each confidence interval were adjusted for the multiplicity of tests using the Bonferroni rule. Column 1: TD-fNIRS parameters (*A* peak amplitude, *τ* = peak latency, *O*_*2*_*Hb* oxy-haemoglobin, *HHb* deoxy-haemoglobin). Column 2: estimated OR (*Est* estimates). Column 3: tests of association between parameters and pathological classification of eyes (*Df* degrees of freedom. *χ*^*2*^ Chi-square statistics. *p < 0.05; **p < 0.01). Column 4: estimates of the concordance index c.

For A_HHb_, the OR estimates range from 3.31 to 12.63 (Table 2), thus indicating an overall positive association. The association was statistically significant only for the measurements in the right hemisphere (p = 0.0174). For A_O2Hb_, the estimated OR, ranging from 0.32 to 0.53, indicate negative association, and is supported by statistically significant test results in every case (p-values ranging from 0.0091 to 0.0458). Concerning the discriminant ability, the estimates of the concordance index for A_O2Hb_ and A_HHb_ range from 0.677 to 0.707, and are significantly greater than 0.5 (no discrimination) in every case, as shown by the respective 95% C.I.s (Table 2). On the contrary, no significant association with pathological classification was found for τ_O2Hb_ and τ_HHb_, and the estimated OR are very close to one (from 0.89 to 1.05). The discriminant ability was not significantly higher than 0.5.

#### HRF amplitudes and latencies recorded at both hemispheres

The results reported in this paragraph were obtained from logistic regression models that include two independent variables, i.e. the parameters recorded at both hemispheres (Table 3). For evaluating the association with the pathological classification we used the following strategy: (1) if no association emerged from the models for parameters recorded at single hemispheres (previous paragraph) a test was performed by setting as null hypothesis the hypothesis that both parameters are equal to zero; (2) otherwise, we considered two Wald tests, one for each parameter in the model. The correction for test multiplicity was then applied using the actual number of tests. Results are reported in Table 3.

**Table 3:** Association and discriminant ability of TD-fNIRS parameters recorded at both hemispheres.

TD-fNIRS parameter	OR Est (95% C.I.)	Wald test			c index Est (95% C.I.)
		Df	χ^2^	p-value	
**τ**_**HHb**_					
Left hemisphere	1.07 (0.91, 1.25)	2	2.690	0.5220	0.553 (0.453, 0.652)
Right hemisphere	0.92 (0.79, 1.06)				
**τ**_**HHb**_					
Ipsilateral hemisphere	1.01 (0.89, 1.15)	2	0.635	> 0.999	0.549 (0.449, 0.650)
Contralateral hemisphere	0.97 (0.85, 1.09)				
**τ**_**O2Hb**_					
Left hemisphere	0.88 (0.73, 1.08)	2	2.470	0.5820	0.583 (0.484, 0.682)
Right hemisphere	1.03 (0.84, 1.26)				
**τ**_**O2Hb**_					
Ipsilateral hemisphere	0.92 (0.76, 1.12)	2	1.200	> 0.999	0.588 (0.489, 0.687)
Contralateral hemisphere	0.98 (0.84, 1.14)				
**A**_**HHb**_					
Left hemisphere	2.78 (0.23, 33.80)	1	1.060	0.9094	0.730 (0.637, 0.823)
Right hemisphere	11.80 (1.07, 129.00)	1	6.694	0.0290**	
**A**_**HHb**_					
Ipsilateral hemisphere	7.97 (0.83, 76.10)	2	8.950	0.0342*	0.725 (0.632, 0.8)
Contralateral hemisphere	4.23 (0.81, 22.30)				
**A**_**O2Hb**_					
Left hemisphere	0.41 (0.12, 1.42)	1	3.250	0.2858	0.712 (0.612, 0.813)
Right hemisphere	0.67 (0.30, 1.47)	1	1.670	0.7870	
**A**_**O2Hb**_					
Ipsilateral hemisphere	0.56 (0.31, 1.04)	1	5.490	0.0763	0.711 (0.610, 0.811)
Contralateral hemisphere	0.50 (0.22, 1.16)	1	4.240	0.1578	

Results obtained by estimating 8 logistic models including the same parameter obtained by the haemodynamic response at two distinct hemispheres, e.g. τ_HHb_ from haemodynamic response recorded at the left hemisphere and τ_HHb_ from haemodynamic response recorded at the right. Each p-value and each confidence interval were adjusted for the multiplicity of tests using the Bonferroni rule. Column 1: TD-fNIRS parameters (*A* peak amplitude, *τ* peak latency, *O*_*2*_*Hb* oxy-haemoglobin, *HHb* deoxy-haemoglobin). Column 2: estimated OR (*Est* estimates). Column 3: tests of association between parameters and pathological classification of eyes (*Df* degrees of freedom. *χ*^*2*^ Chi-square statistics. *p < 005, **p < 0.01). Column 4: estimates of the concordance index c.

For A_HHb_, the estimated OR ranges from 2.78 to 11.80; while for A_O2Hb_ the OR ranges from 0.41 to 0.67. These values are overall comparable with the corresponding estimates, reported in Table 2, that refer to the models for single hemispheres. The estimates of the discriminant ability range from 0.711 to 0.730 and are comparable to the values obtained for single-parameter models (Table 2).

For τ_O2Hb_ and τ_HHb_, no significant association was found. Furthermore, the discriminant ability was overall not significantly greater than 0.5.

#### Differences and ratios of parameters from O_2_Hb and HHb response curves

Simple and absolute value for the differences (τ_O2Hb_ − τ_HHb_) and the ratios (A_O2Hb_/A_HHb_) were calculated, and included in distinct regression models as independent variables (Table 4). Thus, for each of the four pre-defined functions, four tests were performed, and used for correction of test multiplicity. For the simple difference (τ_O2Hb_ − τ_HHb_) no significant association with pathological classification was found, and the discriminant ability was non-significantly greater than 0.5. A significant association was found for the absolute difference |τ_O2Hb_ − τ_HHb_| for records at the left hemisphere (OR 1.27, p = 0.0027). This result suggests that the distance between τ_O2Hb_ and τ_HHb_ latencies could be tendentially higher in glaucomatous eyes as compared to healthy eyes. A significant association was found also for the contralateral hemisphere (absolute difference: OR 1.22; p = 0.0049), with a similar interpretation as above. Furthermore, c index for the absolute differences above was 0.637 and 0.630, even though not significantly higher than 0.5. For what concerns the ratio (A_O2Hb_/A_HHb_), the association was weak (OR ranging from 0.99 to 1.05) and overall not significant.

**Table 4:** Association and discriminant ability of pre-defined functions of TD-fNIRS parameters.

TD-fNIRS parameter	OR Est (95% C.I.)	Wald test			c index Est (95% C.I.)
		Df	χ^2^	p-value	
**τ: difference**					
Left hemisphere	0.89 (0.78, 1.01)	1	5.290	0.0859	0.560 (0.449, 0.672)
Right hemisphere	1.04 (0.92, 1.17)	1	0.731	> 0.999	0.532 (0.417, 0.647)
Ipsilateral hemisphere	0.95 (0.85, 1.05)	1	1.678	0.7809	0.545 (0.433, 0.635)
Contralateral hemisphere	1.01 (0.91, 1.12)	1	0.045	> 0.999	0.518 (0.405, 0.631)
**τ: absolute difference**					
Left hemisphere	1.27 (1.07, 1.52)	1	11.600	0.0027*	0.660 (0.496, 0.823)
Right hemisphere	1.07 (0.91, 1.26)	1	1.020	> 0.999	0.576 (0.450, 0.702)
Ipsilateral hemisphere	1.08 (0.94, 1.25)	1	1.860	0.6880	0.602 (0.466, 0.739)
Contralateral hemisphere	1.22 (1.05, 1.42)	1	10.500	0.0049*	0.637 (0.486, 0.789)
**A: ratio (relative values)**					
Left hemisphere	1.04 (0.99, 1.09)	1	3.760	0.2100	0.628 (0.481, 0.776)
Right hemisphere	1.05 (0.90, 1.22)	1	0.544	> 0.999	0.582 (0.452, 0.711)
Ipsilateral hemisphere	1.04 (0.99, 1.10)	1	4.045	0.1772	0.613 (0.471, 0.755)
Contralateral hemisphere	1.02 (0.95, 1.10)	1	0.671	> 0.999	0.593 (0.461, 0.726)
**A: ratio (absolute values)**					
Left hemisphere	1.01 (1.00, 1.03)	1	4.246	0.1574	0.533 (0.421, 0.645)
Right hemisphere	0.92 (0.80, 1.06)	1	2.383	0.4905	0.573 (0.461, 0.685)
Ipsilateral hemisphere	1.00 (0,97, 1.02)	1	0.057	> 0.999	0.573 (0.462, 0.683)
Contralateral hemisphere	1.01 (0.92, 1.10)	1	0.027	> 0.999	0.534 (0.421, 0.647)

Results obtained by estimating 16 distinct logistic models including a single independent variable, i.e. the differences τ_O2Hb_ − τ_HHb_ and the ratios A_O2Hb_/A_HHb_. Each p-value and each confidence interval was adjusted for the multiplicity of tests, using the Bonferroni rule. Column 1: TD-fNIRS parameters (*A* peak amplitude, *τ* peak latency). Column 2: estimated OR (*Est* estimates). Column 3: tests of association between parameters and pathological classification of eyes (*Df* degrees of freedom. *χ*^*2*^ Chi-square statistics. *p < 0.05). Column 4: estimates of the concordance index c.

The analysis was extended to regression models for differences and ratios relative to both hemispheres (in an analogous fashion to the models in the previous paragraph). The results, reported in Supplementary Table ST3, do not provide any substantial improvement to the interpretation given in this paragraph.

## Discussion

This study investigated the HRF of the occipital cortex following visual stimulations in glaucomatous eyes by means of TD-fNIRS. To our knowledge this is the first TD-fNIRS study on glaucoma eyes, in which we have recorded a sample size substantially larger than previous fNIRS studies.

The TD-fNIRS quality check applied to the collected data determined that data from only 3 subjects had to be completely discarded because of the presence of TD-fNIRS specific issues (e.g. direct light between source and detector or low photons count rate). The limited amount of discarded data indicates that the optical probe was properly designed, guaranteeing a good adhesion to the scalp and, then, the absence of movement artefacts. Furthermore, the high rate of acceptance among the subjects’ population is an evidence of the satisfactory signal-to-noise ratio generally observed.

One of the advantages of TD-fNIRS over continuous wave (CW) fNIRS is the possibility to retrieve the absolute values of the tissue’s optical properties, i.e. absorption (μ_a_) and reduced scattering (μ_s_′) coefficients. We used the average optical properties to estimate the mean photons’ pathlengths inside the tissue for the extracerebral tissue and for the cerebral cortex with a semi-empirical method^18^. This method needs, as input, the thickness of the extracerebral layer. In the work by Lynnerup^19^ a cranial thickness between 5 and 10 mm for the occipital region of adults is reported. In addition, Wendel et al*.*^20^ presented skin thickness values lower than 2.5 mm. Zhao et al*.*^21^ explained that around the inion point, on average, a 15 mm thickness is reported. We stress that in our case, the upper layer thickness has the significance of an equivalent layer, which has to be enough thick to allow to remove the contribution of the capillary bed present in the scalp and in the skull, but also not too thick to eliminate possible contributions of the more superficial part of the cerebral cortex. At this purpose we choose a conservative thickness of 0.5 cm, which is the minimum value we found for the cranial bone in the occipital region.

The TD-fNIRS time courses in healthy eyes (NORM) show the typical haemodynamic cortical activation, i.e. increase in O_2_Hb and a contextual decrease in HHb^22^. From the fitting with a canonical HRF model we were able to estimate, for O_2_Hb and HHb, the amplitude of the response and the delay with respect to the start of the stimulation. Our data on healthy eyes are in line with literature findings for visual stimulation^22^ showing that |A_O2Hb_/A_HHb_|> 1 and τ_O2Hb_ ≈ τ_HHb_. For what concern the glaucomatous patients, there are scarce literature data to compare with. Ward et al*.*^15,23^ considered 8 glaucoma patients during visual stimuli and found an attenuated response, that is a lower A_O2Hb_ than in healthy participants. Our results have confirmed their findings in a larger and more homogeneous group of subjects.

In the present analysis, OR provides a quantitative evaluation of the strength of association between a categorical response variable (pathological classification in our case) and a (categorical or numerical) independent variable: OR > 1 indicates positive association (an eye with a given value of a fNIRS parameter has a higher probability of being glaucomatous as compared to an eye with a lower fNIRS parameter value, and a lower probability with respect to an eye with a higher parameter value), while OR < 1 indicates negative association (an eye with a given value of an fNIRS parameter has a lower probability of being glaucomatous as compared to an eye with a lower fNIRS parameter value, and a higher probability with respect to an eye with a higher parameter value); while an OR 1 indicates weak or no association at all. We estimated OR values lower than one for A_O2Hb_ and OR values higher than one for A_HHb_. This was consistent with the fact that estimated A_O2Hb_ amplitudes tend to be slightly larger in normal than in glaucomatous eyes, and A_HHb_ slightly lower in normal than in glaucomatous eyes. That is equivalent to say that a glaucoma eye, gives rise to a smaller activation in the occipital area, when exposed to visual stimuli (Table 2). On the contrary, OR ≈ 1 for both τ_O2Hb_ and τ_HHb_, showing that the latency has no relevant association with the presence of a glaucomatous eye. The only exception is when τ_O2Hb_ are recorded at the left and ipsilateral hemisphere. This significant τ response in the left hemisphere, although present, has no clear clinical explanation. In literature, the only result that could be somehow similar was previously found by Kashou et al*.*^24^: the authors affirm that typically the left hemisphere has a higher activation with respect to the right one. This phenomenon should be better investigated, taking into the account the association with the predominant hand which could have a higher cortical representation on the opposite hemisphere or to the dominant eye as well.

Furthermore, since the concordance indexes *c* found for the two-hemisphere model (Table 3) are similar to those found for the single parameter analysis, this support the evidence that using the information coming from both the hemispheres would not improve the predictive capacity with respect to using just one hemisphere. Also the use of combined parameters, such as the ratio of the amplitudes or the differences of the latencies, does not show an increase in the association between these parameters and the glaucomatous state of an eye, as shown in Table 4 (OR ≈ 1).

In this study primary open angle glaucoma eyes were considered as one clinical category (GLAUCOMA). There are several studies in literature showing a different involvement of post-retinal visual pathways in hypertensive and normal pressure glaucoma, i.e. at the level of the lateral geniculate nucleus of the thalamus and the primary visual cortex (V1)^25^. On the other hand, ocular hypertensions, i.e. subjects with a clear increase in IOP without papillary and/or visual field alterations, have a rate of conversion over time into manifest glaucoma that differs in different studies and whether or not they undergo hypotonizing therapy. In this context, in a next study we propose three groups for the analysis, i.e. ocular hypertension, hypertensive glaucoma and normal pressure glaucoma.

## Methods

### Ethical statement

The study was approved by the Sacco Hospital Medical Ethical Committee (no. 0018034, 07/07/2015) and by the Ministry of Health (DGDMF.VI/P/I.5.m.i.2/2015/1022) and was conducted in compliance with the Declaration of Helsinki. All subjects gave their written informed consent to participate to the study following an explanation of the nature and possible consequences of the study.

### Subjects

According to the clinical protocol, a total of 98 subjects were chosen and went through a complete clinical examination including: complete ophthalmological inspection, best-corrected visual acuity, Goldmann tonometry, assessment of intraocular pressure (IOP), evaluation of the ocular fundus with quantification of papillary excavation (cup disk ratio), computerized visual field (evaluation of perimetric indexes Mean defect and corrected loss variance), electrophysiological examinations (visual evoked potentials and pattern electroretinogram), optic nerve head assessment Optical Coherence Tomography examination of the papilla (evaluation of peripapillary and macular ganglion cells, by sections). Inclusion criteria were: visual acuity of 15/20 natural or with refractive correction; refractive error between ± 3 spherical diopters and ± 1 cylindrical diopters; IOP less than 20 mmHg, already in medical therapy with antiglaucomatous topical eye drops; typical glaucomatous changes in the optic disk. Exclusion criteria were: endocular surgery performed in the last three months; other ocular and/or systemic pathologies. Based on these criteria, 4 subjects were excluded from the study and did not took part to the TD-fNIRS acquisition sessions.

According to the standard tests, the subjects were classified as healthy (NORM) and glaucoma patients (GLAUCOMA) with a structural damage at the optic nerve head and visual field loss. Primary open-angle glaucoma were included and narrow-angle glaucoma and secondary glaucomatous eyes were excluded. Subjects with open-angle glaucoma were included in the study with these features: a mean defect (MD) and pattern standard deviation with p < 0.05 probability of being normal; a pattern standard deviation probability of < 5%; or a cluster of three or more adjacent non-edge points in typical glaucomatous locations that did not cross the horizontal meridian, all of which were depressed on the pattern deviation plot at P < 5%, and 1 of which was depressed at P < 1%. The glaucomatous visual field loss had to be confirmed in at least 2 consecutive tests. During the enrollment of the subjects, since it was demonstrated that the fNIRS baseline haemodynamic parameters have changes according to the subjects age^26^, an age matched NORM group with the GLAUCOMA one was chosen.

### Acquisition protocol

The acquisition protocol was designed as: 30 s of initial baseline followed by 5 repeated stimulation cycles (10 s rest, 10 s visual stimulus, 10 s recovery), for a total length of 180 s. The protocol was repeated for each eye. Each subject sat in a dimly light room, in front of a monitor at a distance of 130 cm and had to fix its center with one eye at a time (the other eye was closed with a shutter). According with previous literature findings^27^, the chosen visual stimulus was a pattern reversal checkerboard (viewing angle 30′, 1.13 cm length) with a reversal frequency of 10 Hz. The rest and recovery screens were grey, with an equivalent luminance to the checkerboard stimulus. The stimuli were presented on a computer screen using the software Presentation (Neurobehavioral Systems Inc., Albany, CA) and were synchronized with the TD-fNIRS device acquisitions with a TTL signal. The head circumference, and the landmarks on the skull (left and right preauricular, nasion and inion) were measured for each subject before the acquisition, in order to find the best EEG cap size and its placement on the head. The source-detector distance was 3 cm. For each subject an instrument response function (IRF) was also acquired^28^.

### TD-fNIRS data acquisition

To perform TD-fNIRS acquisitions, the multi-channel medical device approved by the Italian Ministry of Health previously developed by the authors at the Department of Physics at Politecnico di Milano, was employed^29^. It is based on two pulsed diode lasers operating at 687 and 826 nm as light sources and four hybrid photomultipliers tubes as detectors. Laser pulses are delivered to the tissue by means of optical fibers according to the space multiplexing modality^30^. The electronic acquisition chain is based on time correlated single photon counting technique. For further details, the reader can refer to the paper by Re et al*.*^29^.

**Figure 4:**
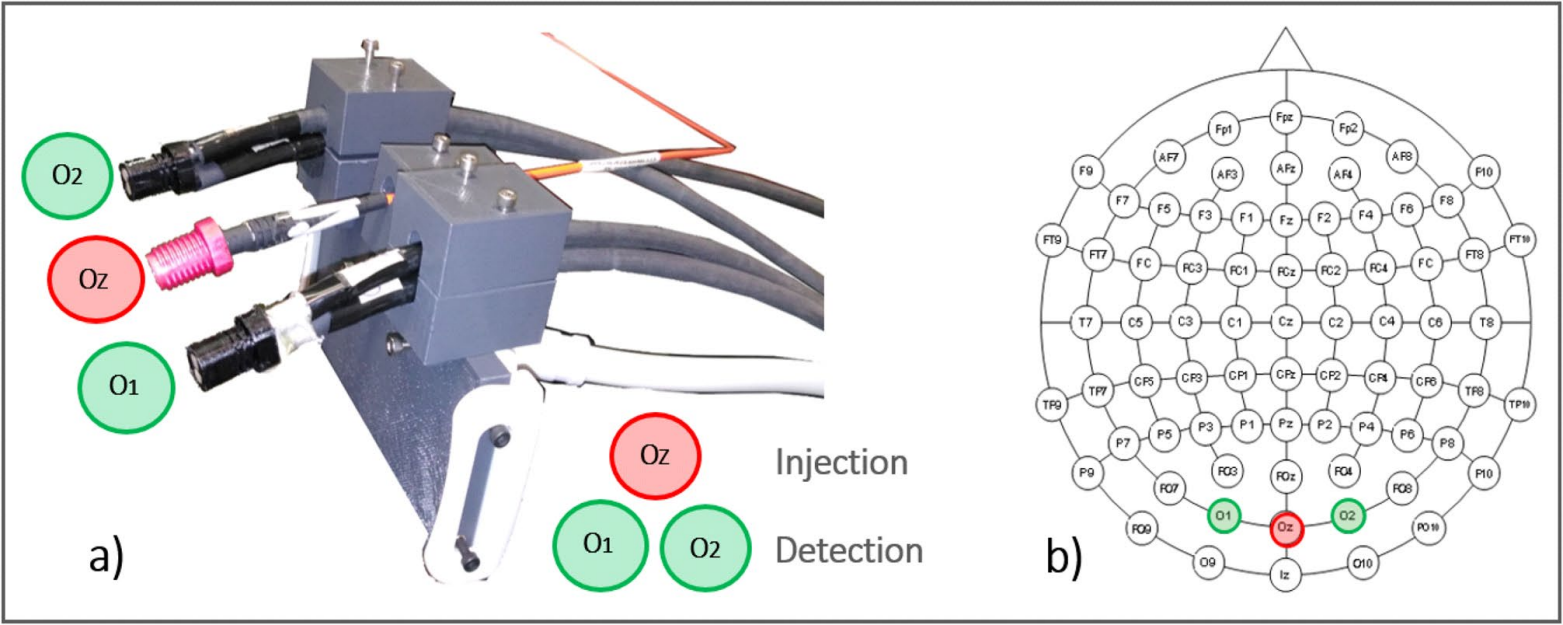
(**a**) Optical probe picture. (**b**) Optodes positioning on the occipital region according to the 10–10 EEG system^17^. Oz: injection optode. O1 and O2: detection optodes.

To facilitate the acquisition from the back of the head (occipital region) a custom support for the optical fibers was developed, assembling a commercial support for smartphone and a custom 3D printed part. It was printed with a filament printer (Sharebot NG, Sharebot s.r.l., Nibionno, Italy) and a PLA filament (3DiTALY, Roma, Italy)^31^. In Fig. 4a, a picture of the optical probe is shown. As a tradeoff between complexity of setting the probe on the subjects and need of collecting response form the occipital area, we choose a simple arrangement with one injection (in the OZ position) and 2 detection optodes (on O1 and O2 positions, respectively)^24,32^. In Fig. 4b the optodes positioning is schematically represented. The optical fibers were placed on the subject’s head by means of a standard EEG cap and fixed in the cap holes with a proper custom system which was able to maintain a good adhesion during the whole acquisitions.

### TD-fNIRS data analysis

The TD-fNIRS signals (photon distributions of time-of-flight) acquired, at both wavelengths and for each acquisition channel, were fitted with the solution of the diffusion equation for a semi-infinite homogeneous medium, after being convolved with the IRF^33^. We checked the acquisitions’ quality in terms of detected photons number (> 150 kcounts in the initial baseline) and fitting parameter (χ^2^ ≤ 2). In this way, absolute values for the absorption and reduced scattering coefficients were retrieved during the baseline period. From these values the photons’ pathlengths in a two-layer medium, mimicking the extra-cerebral and the cerebral tissue, were calculated and used to estimate the variations of the cortical O_2_Hb and HHb concentration as described in Zucchelli et al*.*^18^. This method allows for discriminating photons which have travelled more in depth (cerebral cortex) from those ones which had a higher probability to have travelled more through the surface (skull and scalp), reducing the confounding effect of systemic changes on the fNIRS signal. A thickness for the superficial layer of 0.5 cm was chosen.

The O_2_Hb and HHb concentration time courses were then fitted with a canonical haemodynamic response function (HRF)^34^. From the fitted curves, we then determined for each repeated stimulation: the response amplitudes A_O2Hb_ and A_HHb_, for O_2_Hb and HHb respectively, and response latencies with respect to the stimulus onset τ_O2Hb_ and τ_HHb_, for O_2_Hb and HHb respectively.

### Statistical analysis

The main goal of the statistical analysis was to evaluate the ability of discriminating between healthy and glaucomatous eyes using the fNIRS parameters A_O2Hb_, A_HHb_, τ_O2Hb_, and τ_HHb_. Thus, for these analyses we considered the single eye as sample unit, rather than the subject. Data consisted of pathological classifications of eyes, the respective records of A_O2Hb_, A_HHb_, τ_O2Hb_, and τ_HHb_ and the following experimental factors: eye (left, right), hemisphere (left, right) and stimuli (1 to 5). The distributions of the values observed for the different parameters were summarized separately for healthy and glaucomatous conditions according to the experimental factors, using medians and quartiles, and graphically represented using boxplots. For each parameter, the relationships with the pathological classifications were assessed by logistic regression models with binary response variable (assuming the values: 1 for GLAUCOMA eyes, 0 for NORM eyes). As independent variables we used the mean values of parameters at hemisphere level, that is, the means across the responses to the five stimuli recorded at a single hemisphere. We looked for putative differences between healthy and glaucomatous condition related to brain side (left/right) and to the relative position between hemispheres and eyes (ipsilateral/contralateral). To such aim, several logistic models including mean parameters of the haemodynamic responses were fitted, using two hemisphere classifications: left/right and ipsilateral/contralateral. Lastly, we investigated putative differences between healthy and glaucomatous condition related to relationships between HRF for O_2_Hb and HHb. In this case, we used logistic models including specific functions of the means of the parameters above: simple and absolute values for the difference (τ_O2Hb_ − τ_HHb_) and for the ratio (A_O2Hb_/A_HHb_). The simple difference (τ_O2Hb_ − τ_HHb_) is a measure of the time shift of the peak of the O_2_Hb curve respective to the peak of the HHb curve, while the absolute value |τ_O2Hb_ − τ_HHb_| provides a measure of the lag between the two peaks without accounting for which one occurs first. In an analogous fashion, the ratio (A_O2Hb_/A_HHb_) accounts for the sign of the signal, whereas the |A_O2Hb_/A_HHb_| considers only the magnitude of the peaks. For each of the previously mentioned models, the association of the fNIRS parameters with eye’s pathological classification was evaluated through the estimated Odds Ratio (OR) with respective 95% confidence intervals (C.I.), and by the Wald test (Chi-square distribution). The C.I.s and the p-values were corrected for test multiplicity, using the Bonferroni rule. For each parameter the probability of type I error (nominal level of 5%) was corrected by the number of Wald tests performed.

The discriminating ability of fNIRS parameters (primary endpoint) was assessed using the concordance index *c*^35^, which is equivalent to the area under the Receiver Operating Characteristic (ROC) curve for discriminating healthy and glaucomatous eyes. The discriminant ability was deemed statistically significant when the confidence intervals of the concordance index did not include the value of 0.5 (worst discriminant ability as defined by ROC theory). The Bonferroni rule was adopted for correcting the confidence intervals for the c index, using the same procedure previously described.

In order to account for the correlation among measurements obtained at fellow eyes, which leads to the violation of the i.i.d. sampling assumption, the models were estimated by the Generalized Estimating Equations method^36^. The analysis was performed with the software R release 3.5.1^37^ with the package geepack^38^ added, and KNIME analytics platform release 4.0.1^39^.

## Conclusion

The main objectives of this pilot study were to evaluate the possible involvement of the visual occipital cortical areas for glaucomatous eyes and to verify whether this aspect could be detected by TD-fNIRS. From the TD-fNIRS analysis we estimated the latencies and the amplitudes of the HRF following a visual stimulation. Our findings showed that the amplitude of O_2_Hb and HHb concentrations in the glaucomatous eyes are lower than in the control group. These results suggest that in glaucomatous patients there could be a multi-level neuronal degeneration of the visual pathways, including the occipital cortical region. Moreover, the easiness of use of the TD-fNIRS technique, makes this optical technique a good candidate for a better understanding of the glaucoma pathology. It was not feasible to perform an analysis using patients as statistical unit instead of eyes, because of the heterogeneity of HRF responses for the fellow eyes, thus criteria to integrate the responses of the two hemispheres are lacking in our case. Furthermore, due to the presence of mixed patients in clinical routine, a topic to be investigated consists in how the role of degenerated visual pathways could influence the hemodynamic responses. However, to such end a higher sample size (in particular a higher number of mixed patients) and a wider experimental design are needed, including a more extensive coverage of the occipital/brain area.

As future perspectives, in the glaucoma context, it could be of interest to study the baseline optical properties of patients since structural differences between healthy subjects and glaucomatous patients were underlined in some neuroimaging studies. Subjects with advanced glaucoma can show visual field alteration in corresponding location of the flattened cortex^14^. Moreover, they show grey matter atrophy both in visual system and in non-visual brain regions, without evident differences^40^. Further, there are more diffuse cerebral small-vessel ischemic changes in the glaucoma patients than in controls^41^. Interestingly, it was demonstrated that structural differences between these two groups can be found not only in the occipital region, but also in the frontal cortex^42^, more easily accessible by fNIRS thanks to absence of hairs limiting the signal-to-noise-ratio. Indeed, we found no significant differences in the optical properties of healthy subjects and glaucoma patients. Since we used a homogeneous model, the retrieved optical properties do not refer to the cerebral cortex only, but to the whole volume under the probes. To appreciate fine differences in the neuroanatomical configuration and to discriminate between the contributions coming from more superficial layers (scalp and skull) and the brain cortex, it would be needed to apply more sophisticated acquisition schemes (e.g. multi-distance, broadband) and proper physical models such as a photon diffusion in a two-layer medium or numerical approaches based on finite element method or Monte Carlo scheme in heterogeneous media.

Another interesting aspect, which will be the scope of a following paper, is a careful combined analysis of all the ocular and TD-fNIRS parameters.

## Supplementary Information

Supplementary Information.
