# Supplementary material for: Monitoring the haemodynamic response to visual stimulation in glaucoma patients
Source: Sci Rep. 2021 Jun 30;11:13567. doi: 10.1038/s41598-021-92857-x (PMC8245402; doi:10.1038/s41598-021-92857-x)
Supplement: Supplementary file 1 — Supplementary Information. [file 41598_2021_92857_MOESM1_ESM.docx]

**Supplementary data**

**Monitoring the haemodynamic response to visual stimulation in glaucoma patients**

# R. Re^1,2^*^†^, D. Messenio^3†^, G. Marano^4†^, L. Spinelli^2^, I. Pirovano^1,5^, D. Contini^1^, R. Colombo^3^, P. Boracchi^4^, E. Biganzoli^4,6^, R. Cubeddu^1^, and A. Torricelli^1,2^

^1^Dipartimento di Fisica, Politecnico di Milano, Piazza Leonardo da Vinci 32, 20133 Milan, Italy

^2^Istituto di Fotonica e Nanotecnologie, Consiglio Nazionale delle Ricerche, Piazza Leonardo da Vinci 32, 20133 Milan, Italy

^3^Eye Clinic, Department of Clinical Sciences, ASST Fatebenefratelli Sacco Hospital, University of Milan, Milan, Italy

^4^Laboratorio di Statistica Medica, Biometria ed Epidemiologia “G.A. Maccacaro”, Dipartimento di Scienze Cliniche e di Comunità, Università degli Studi di Milano, Via Vanzetti 5, Milan, Italy

^5^Istituto di Tecnologie Biomediche, Consiglio Nazionale delle Ricerche, via Fratelli Cervi 93, 20090, Segrate (MI), Italy

^6^Unità di Statistica Medica, Biometria e Bioinformatica, Fondazione IRCCS Istituto Nazionale dei Tumori di Milano, Via Vanzetti 5, Milan, Italy

*rebecca.re@polimi.it

^†^these authors contributed equally to this work

**Supplementary Table ST1**. Demographic information of the 86 subjects included in the statistical analysis

|  | **NORMAL**  **(n=31)** | **GLAUCOMA**  **(n=47)** | **MIXED**  **(n=12)** | **OVERALL**  **(n=86)** |
| --- | --- | --- | --- | --- |
| **Gender, Female: n (%)** | 17 (54.8%) | 19 (44.2%) | 6 (50.0%) | 42 (48.8%) |
| **Age at measurement date**  **years: mean ± sd** | 66.0 **±** 8.9 | 68.2 **±** 10.1 | 68.2 **±** 8.7 | 67.4 **±** 9.4 |

**Supplementary Table ST2.** Median and quartiles (Q_1_, Q_3_) of TD-fNIRS parameters

|  | **NORM** | | **GLAUCOMA** | |
| --- | --- | --- | --- | --- |
|  | **LEFT EYE** | **RIGHT EYE** | **LEFT EYE** | **RIGHT EYE** |
|  | **τ_HHb_ [s]** | | | |
| **LEFT HEMISPHERE**  cycle: 1  cycle 2  cycle 3  cycle 4  cycle 5 | 3.1 (1.2, 5.8)  4.7 (2.8, 7.1)  5.0 (3.5, 7.8)  5.1 (2.8, 7.3)  5.0 (2.6, 7.1) | 5.2 (2.9, 6.7)  5.0 (3.5, 7.0)  6.1 (3.8, 7.4)  6.0 (3.4, 7.2)  5.4 (2.8, 7.4) | 4.7 (1.7, 8.6)  4.1 (1.1, 7.3)  4.7 (1.5, 6.8)  5.4 (2.4, 8.4)  5.3 (2.1, 8.8) | 4.3 (2.1, 7.2)  4.7 (1.7, 6.8)  5.1 (2.2, 7.0)  4.6 (2.8, 7.1)  4.6 (1.5, 6.0) |
| **RIGHT HEMISPHERE**  cycle: 1  cycle 2  cycle 3  cycle 4  cycle 5 | 3.1 (1.5, 5.4)  5.7 (2.2, 7.6)  5.3 (2.4, 7.2)  5.2 (3.1, 7.6)  5.4 (3.6, 6.9) | 4.3 (2.3, 8.3)  5.1 (3.3, 6.2)  6.3 (3.4, 7.1)  6.3 (4.5, 8.2)  5.9 (2.7, 7.3) | 6.1 (2.2, 8.3)  5.5 (2.0, 7.4)  4.0 (2.1, 6.7)  4.1 (0.1, 7.2)  4.1 (1.1, 7.1) | 3.9 (2.3, 6.4)  5.3 (1.8, 6.8)  5.4 (1.9, 7.1)  6.5 (2.6, 8.3)  5.1 (3.9, 6.9) |
|  | **τ_O2Hb_ [s]** | | | |
| **LEFT HEMISPHERE**  cycle: 1  cycle 2  cycle 3  cycle 4  cycle 5 | 4.2 (2.4, 6.3)  4.7 (3.5, 7.2)  4.2 (2.2, 6.9)  4.9 (2.8, 6.8)  5.9 (2.6, 6.9) | 4.0 (2.4, 6.0)  4.3 (3.0, 6.5)  5.2 (4.0, 6.5)  5.7 (3.9, 6.7)  5.3 (3.2, 6.8) | 3.7 (1.5, 6.3)  4.0 (1.7, 7.2)  3.0 (1.4, 6.8)  2.0 (0.0, 4.5)  5.3 (2.2, 7.3) | 3.2 (1.2, 6.1)  3.4 (1.6, 5.7)  4.2 (1.5, 6.7)  4.6 (2.8, 7.5)  5.0 (3.5, 7.0) |
| **RIGHT HEMISPHERE**  cycle: 1  cycle 2  cycle 3  cycle 4  cycle 5 | 4.2 (2.7, 5.9)  4.9 (3.6, 6.6)  4.0 (3.2, 5.6)  5.7 (3.6, 7.1)  4.5 (3.1, 6.1) | 5.4 (3.3, 7.8)  4.5 (3.6, 5.3)  4.6 (2.5, 6.3)  4.8 (3.6, 6.5)  5.4 (4.0, 6.8) | 5.3 (3.1, 7.6)  5.5 (3.3, 8.1)  4.0 (1.9, 5.5)  3.2 (0.7, 4.9)  4.1 (2.0, 6.5) | 3.3 (1.8, 6.0)  4.9 (3.0, 7.0)  4.0 (1.3, 6.5)  4.3 (2.4, 7.5)  4.9 (3.6, 6.9) |
|  | **A_HHb_ [μM]** | | | |
| **LEFT HEMISPHERE**  cycle: 1  cycle 2  cycle 3  cycle 4  cycle 5 | -0.18 (-0.25, 0.01)  -0.23 (-0.32, -0.13)  -0.26 (-0.39, -0.18)  -0.17 (-0.42, -0.09)  -0.19 (-0.37, -0.09) | -0.18 (-0.30, -0.09)  -0.21 (-0.35, -0.15)  -0.29 (-0.40, -0.15)  -0.16 (-0.34, -0.09)  -0.25 (-0.37, -0.10) | -0.13 (-0.25, 0.02)  -0.07 (-0.20, 0.05)  -0.14 (-0.24, -0.05)  -0.11 (-0.21, 0.06)  -0.12 (-0.24, 0.04) | -0.09 (-0.19, -0.01)  -0.14 (-0.25, -0.08)  -0.12 (-0.24, -0.07)  -0.13 (-0.25, -0.00)  -0.14 (-0.28, -0.04) |
| **RIGHT HEMISPHERE**  cycle: 1  cycle 2  cycle 3  cycle 4  cycle 5 | -0.15 (-0.31, -0.03)  -0.20 (-0.35, -0.00)  -0.28 (-0.32, -0.16)  -0.28 (-0.45, -0.16)  -0.18 (-0.51, -0.10) | -0.17 (-0.33, -0.05)  -0.21 (-0.34, -0.14)  -0.23 (-0.56, -0.15)  -0.29 (-0.48, -0.12)  -0.27 (-0.44, -0.16) | -0.11 (-0.26, -0.06)  -0.13 (-0.20, -0.05)  -0.15 (-0.23, -0.06)  -0.09 (-0.22, -0.02)  -0.09 (-0.29, -0.03) | -0.14 (-0.20, -0.08)  -0.13 (-0.26, -0.04)  -0.15 (-0.23, -0.06)  -0.21 (-0.29, -0.09)  -0.21 (-0.35, -0.13) |
|  | **A_O2Hb_ [μM]** | | | |
| **LEFT HEMISPHERE**  cycle: 1  cycle 2  cycle 3  cycle 4  cycle 5 | 0.53 (0.26, 0.92)  0.54 (0.30, 1.09)  0.66 (0.40, 1.03)  0.46 (0.34, 1.03)  0.70 (0.27, 1.04) | 0.57 (0.24, 0.75)  0.61 (0.25, 0.83)  0.64 (0.32, 1.10)  0.70 (0.37, 0.87)  0.70 (0.17, 1.05) | 0.24 (-0.09, 0.47)  0.22 (0.08, 0.61)  0.23 (0.08, 0.54)  0.16 (-0.11, 0.45)  0.43 ( 0.18, 0.62) | 0.44 (0.18, 0.61)  0.35 (0.17, 0.73)  0.35 (0.14, 0.65)  0.45 (0.16, 0.68)  0.55 (0.16, 0.71) |
| **RIGHT HEMISPHERE**  cycle: 1  cycle 2  cycle 3  cycle 4  cycle 5 | 0.60 (0.37, 0.95)  0.68 (0.41, 1.38)  0.63 (0.38, 0.97)  0.66 (0.39, 1.38)  0.73 (0.36, 1.18) | 0.58 (0.33, 0.92)  0.57 (0.26, 0.95)  0.62 (0.40, 0.96)  0.59 (0.40, 0.98)  0.77 (0.48, 1.00) | 0.25 (-0.07, 0.53)  0.35 (0.13, 0.68)  0.34 (0.19, 0.62)  0.26 (0.10, 0.58)  0.34 (0.10, 0.61) | 0.39 (0.17, 0.75)  0.46 (0.15, 0.78)  0.37 (0.09, 0.75)  0.41 (0.20, 0.67)  0.55 (0.37, 0.75) |

**Supplementary Table ST3.** Association and discriminant ability of TD-fNIRS parameters recorded at both hemispheres. Column 1: estimates of coefficients of logistic regression models corresponding to TD-fNIRS parameters. Column 2: tests of association between parameters and pathological classification of eyes. Column 3: estimates of the concordance index c. Est= estimates. Df = Degrees of freedom. χ^2^ = Chi-square statistic. *: p<0.05. Each p-value and each confidence interval were adjusted for the multiplicity of tests, using the Bonferroni rule.

| **Parameter** | **OR**  **Est (95% C.I.)** | **Wald test**  **Df** χ**^2^ p-value** | **c index**  **Est (95% C.I.)** |
| --- | --- | --- | --- |
| **τ: difference**  Left hemisphere  Right hemisphere | 0.87 (0.76, 1.00)  1.07 (0.94, 1.21) | 2 5.899 0.1047 | 0.578 (0.479, 0.677) |
| **τ: difference**  Ipsilateral hemisphere  Contralateral hemisphere | 0.95 (0.85, 1.05)  1.01 (0.91, 1.12) | 2 1.704 0.8532 | 0.546 (0.446, 0.646) |
| **τ: absolute difference**  Left hemisphere  Right hemisphere | 1.27 (1.06, 1.53)  1.00 (0.85, 1.18) | 1 10.500 0.0047 *  1 0.001 >0.999 | 0.660 (0.555, 0.765) |
| **τ: absolute difference**  Ipsilateral hemisphere  Contralateral hemisphere | 1.07 (0.93, 1.22)  1.21 (1.04, 1.42) | 1 1.300 >0.999  1 9.760 0.0071 * | 0.643 (0.538, 0.749) |
| **A: ratio**  Left hemisphere  Right hemisphere | 1.03 (0.99, 1.08)  1.04 (0.93, 1.17) | 2 4.169 0.2487 | 0.626 (0.528, 0.724) |
| **A: ratio**  Ipsilateral hemisphere  Contralateral hemisphere | 1.05 (0.98, 1.13)  1.03 (0.95, 1.13) | 2 3.930 0.2803 | 0.626 (0.527, 0.724) |
| **A: ratio (absolute value)**  Left hemisphere  Right hemisphere | 1.02 (0.99, 1.04)  0.92 (0.80, 1.05) | 2 8.052 0.0357 * | 0.591 (0.492, 0.690) |
| **A: ratio (absolute value)**  Ipsilateral hemisphere  Contralateral hemisphere | 1.00 (0.97, 1.02)  1.01 (0.92, 1.10) | 2 0.125 >0.999 | 0.503 (0.402, 0.605) |
